# Benign Feminizing Adrenal Tumor in an Adult Male

**DOI:** 10.31486/toj.19.0031

**Published:** 2020

**Authors:** Raza M. Ahmad, Kyle Ingram, Ralph Corsetti

**Affiliations:** ^1^ The University of Queensland Faculty of Medicine, Ochsner Clinical School, New Orleans, LA; ^2^ Department of Surgery, Ochsner Clinic Foundation, New Orleans, LA

**Keywords:** *Adrenocortical adenoma*, *estrogen-secreting tumor*, *feminizing adrenal tumor*

## Abstract

**Background:** Feminizing adrenal tumors are rare and generally malignant tumors usually seen in male adults and children. We report the case of a benign feminizing adrenal tumor in a male patient. To our knowledge, only 2 other cases of benign, estrogen-only–secreting adrenal tumors have been reported.

**Case Report:** A 44-year-old male with a history of hypertension presented to his primary care physician with chest tenderness, fatigue, and erectile dysfunction. Hormonal workup and imaging identified an estrogen-only–secreting adrenal adenoma. The adenoma was removed via laparoscopic adrenalectomy, and the patient had a normal postoperative course. Pathologic findings were an adrenal cortical adenoma with a Weiss score of 0 and a Ki-67 score of 0%. At 6-month follow-up, the patient's symptoms had significantly improved, and his previously abnormal sex hormone levels were within normal limits.

**Conclusion:** Given the ambiguity in distinguishing between malignant and benign feminizing adrenal tumors, we suggest that radiologic (via Hounsfield units), clinical (via trending hormone levels), and histopathologic (via Weiss and Ki-67 scores) findings are sufficient to confirm the benign nature of this commonly malignant tumor.

## INTRODUCTION

Feminizing adrenal tumors are extremely rare adrenal neoplasms. Chentli et al identified 50 cases reported between 1979 and 2014.^1^ Forty-one patients were males, 33 were adults, and all but 1 case were malignant. In a study conducted by Moreno et al, feminizing adrenal tumors accounted for 0.37% of 801 adrenalectomies between 1970 and 2003, and all of them were malignant.^2^ The most common initial presenting symptoms in males are gynecomastia, erectile dysfunction, and fatigue.^1^ Gynecomastia is usually bilateral and painful, and the size varies significantly among patients. This breast development is related to the high levels of estrogen being secreted from the tumor via increased activity of aromatase and the peripheral conversion of androstenedione to estrone.^3^ Estrogen production can be solitary but is more commonly accompanied by the production of other adrenocortical hormones.

The prognosis of patients with malignant tumors varies in the literature, with Chentli et al reporting a survival of 4 years, Moreno et al reporting a survival of 7 years, and Lanigan et al reporting a median survival of 2.9 months.^1,2,4^ All of the tumors in these 3 studies showed aggressive pathology and were considered carcinomas according to the consensus guidelines (requiring a Weiss score >2).^5^

We report a case of benign feminizing adrenal tumor in a male patient. To our knowledge, only 2 other cases of benign estrogen-only–secreting adrenal tumors have been reported.^6,7^

## CASE REPORT

A 44-year-old male with a history of hypertension presented to his primary care physician in January 2017 for chest tenderness, predominantly on the left, for the prior several months. He had also noticed an increase in the size of both breasts. The patient was an active individual with a regimen of weightlifting and basketball 3 times per week, but he reported weight gain (principally central adiposity) and feeling fatigued. Additionally, he had had erectile dysfunction symptoms since 2016 for which he had seen a urologist. Mammogram revealed benign bilateral gynecomastia that was managed conservatively.

**Figure 1.**
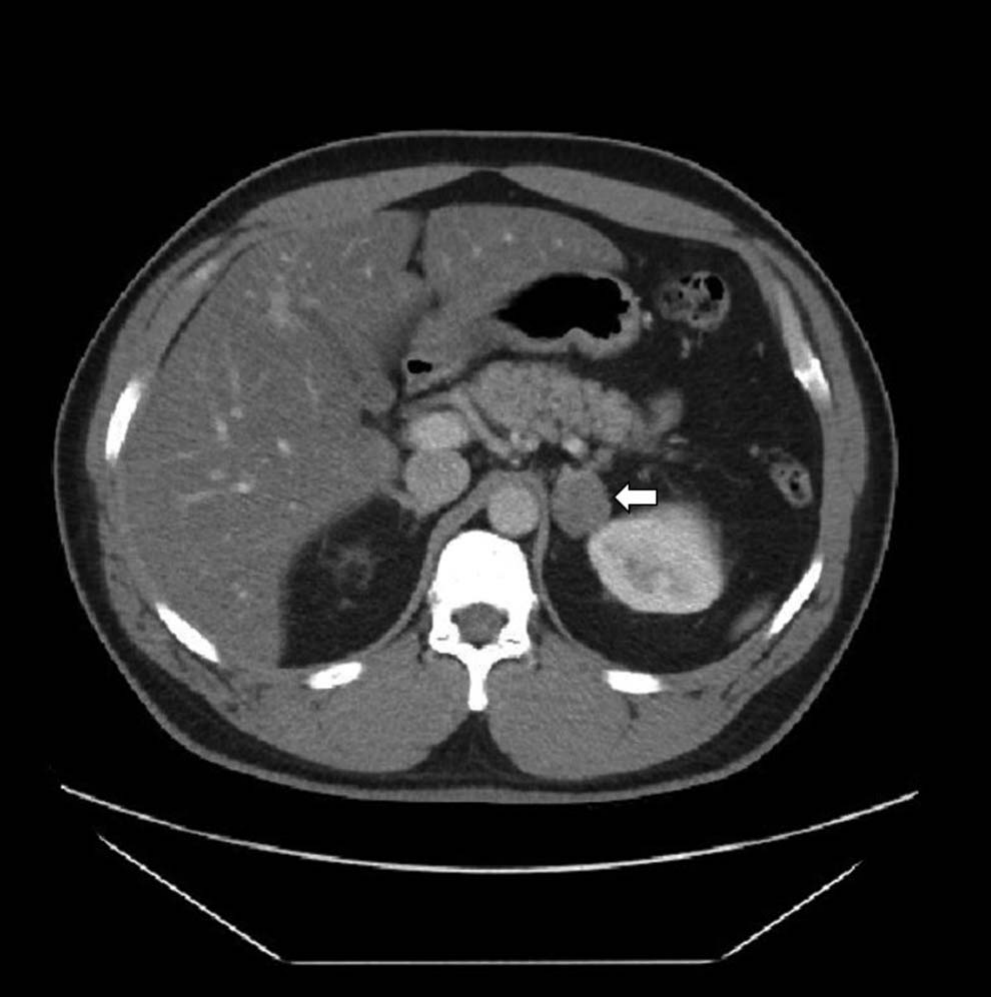
**Computed tomography scan of abdomen and pelvis with contrast shows interval development of a 2.8-cm left adrenal nodule with imaging characteristics consistent with an adenoma (arrow).**

In January 2018, he presented again to his primary care physician with spontaneous improvement of the previously reported chest pain but continued symptoms of fatigue and erectile dysfunction. He was referred to an endocrinologist who determined that his breasts were still enlarged but overall had not changed since his previous mammogram. Hormonal workup showed an elevated estrone level of 192 pg/mL (male reference, <68 pg/mL), elevated estradiol of 44 pg/mL (male reference, <29 pg/mL), low follicle-stimulating hormone of 0.8 mIU/mL (male reference range, 1.6-8.0 mIU/mL), and low testosterone of 28 ng/dL (male reference range, 250-827 ng/dL). Human chorionic gonadotropin and alpha-fetoprotein levels were normal, and testicular ultrasounds to evaluate for testicular and extragonadal germ cell tumors were unremarkable. Pituitary hormonal workup to evaluate secondary hypopituitarism was normal (Table 1). Adrenal computed tomography (CT) revealed a left 2.8-cm nodule that was not visible on a CT done in 2009 (Figure 1). On noncontrast CT, the Hounsfield unit value for the nodule was 10. On contrast CT, the nodule had a washout >50%, with calculation of absolute and relative washout consistent with an adenoma. Adrenal intermediaries checked to evaluate for malignancy were also normal, as was a workup for hypercortisolism (Cushing syndrome) (Tables 2 and 3).

**Table 1.** Pituitary Hormonal Workup

Test	Result	Reference Range
Prolactin, ng/mL	10.3	2.0-18.0
Luteinizing hormone, mIU/mL	3.1	1.5-9.3
Follicle-stimulating hormone, mIU/mL	0.8	1.6-8.0
Thyroid-stimulating hormone, mIU/L	2.08	0.4-4.5
Thyroxine, ng/dL	1.3	0.8-1.8
Plasma renin, ng/mL/h	0.75	0.6-4.3

**Table 2.** Adrenal Intermediaries Workup

Test	Result	Reference Range
Androstenedione, ng/dL	96	40-190
Dehydroepiandrosterone sulfate, mcg/dL	173	70-495
17-ketosteroids, mg/24 h	10.7	5.3-17.6
Deoxycorticosterone, ng/dL	42	10-79
Progesterone, ng/mL	<0.5	<1.4
17-hydroxyprogesterone, ng/dL	181	33-195
Metanephrines, free, pg/mL	40	<57
Metanephrines, total urine, pg/mL	82	44-261

**Table 3.** Hypercortisolism (Cushing Syndrome) Workup

Test	Result	Reference Range
Urinary free cortisol, mcg/24 h	19	3.5-45
Cortisol, 8:00 am, mcg/dL	18.8	10-20
Adrenocorticotropic hormone, pg/mL	12	6-50
Dehydroepiandrosterone sulfate, mcg/dL	173	70-495

**Figure 2.**
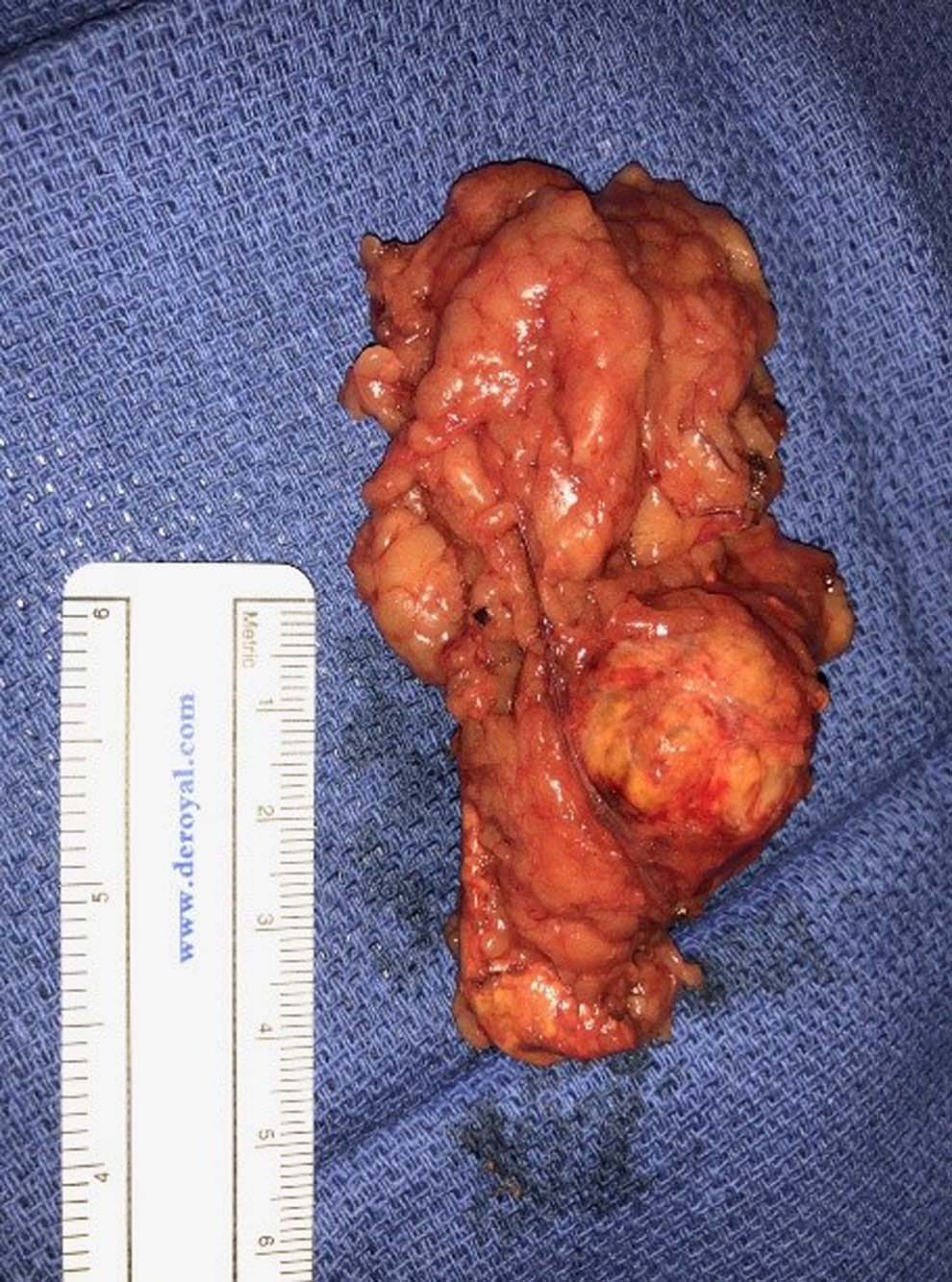
**The left adrenal gland and adenoma have benign macroscopic features such as encapsulation and a homogenous appearance.**

**Figure 3.**
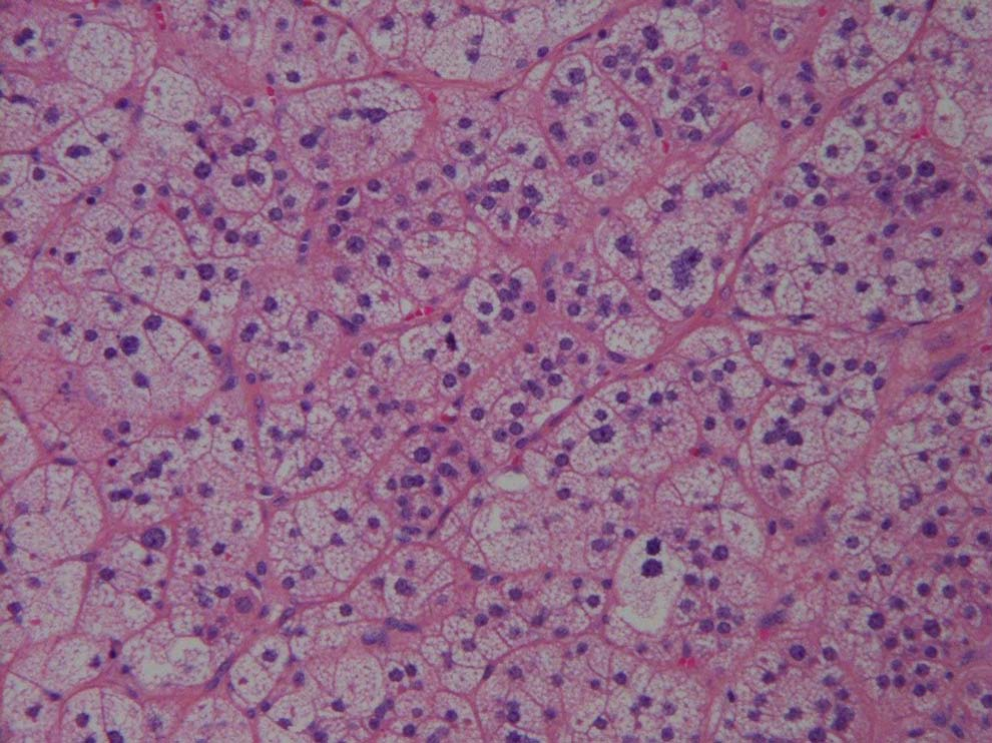
**Features of the microscopic image (hematoxylin and eosin stain, 50×) of the adrenal adenoma are consistent with a benign appearance: <1 mitotic figure per 50 high power fields, no atypical mitotic figures, no necrosis, clear cells >25%, and a modified Weiss score of 0.**

The patient underwent outpatient laparoscopic left adrenalectomy (Figure 2) in June 2018. He had no adverse events during the procedure and was discharged the same day. Pathology was reported as an adrenal cortical adenoma with a Weiss score of 0 and a Ki-67 score of 0% (Figure 3). At 2- and 6-month follow-up, the patient had no complaints of breast tenderness or other symptoms, and his previously abnormal hormonal levels were within normal limits.

## DISCUSSION

The paucity of data describing truly benign feminizing adrenal tumors leads to difficulties in distinguishing between benign and malignant tumors. Mongiat-Artus et al postulated that any adrenal tumor secreting estrogens should be considered malignant regardless of presentation and size.^8^ However, current consensus guidelines consider the components of the Weiss score—mitotic rate, atypical mitoses, high nuclear grade, low percentage of clear cells, necrosis, diffuse architecture of tumor, capsular invasion, sinusoidal invasion, and venous invasion—as essential in distinguishing between benign and malignant.^5^ Another important aspect to consider is the proliferative indices, such as Ki-67, as these values are significantly higher in the setting of adrenocortical carcinoma vs adenoma. Beuschlein et al suggested a grading system in relation to Ki-67 in which higher Ki-67 scores are associated with higher tumor grades and worse prognoses.^9^ A Ki-67 score <10% is considered grade 1, scores between 10% and 19% are grade 2, and scores >20% are grade 3. The feminizing adrenal tumors presenting with high mitotic rates (a component of the Weiss score) were associated with a dire prognosis.^5^ We used the Weiss score and the Ki-67 score to label our case as benign and suggest that if a tumor is radiologically, macroscopically, and histopathologically diagnosed as benign with Hounsfield units, Weiss score, and Ki-67 score, the tumor should be considered benign, regardless of the patient's hormonal status.

As mentioned previously, only 2 other cases of benign estrogen-only–secreting adrenal tumors have been reported, and only 1 was in a male patient with feminizing features as in our case.^6,7^ The outcome of the male patient—reported by Andía Melero et al—was not expected, as 3 years postadrenalectomy, he had a reoccurrence with metastasis and died.^7^ This patient had cellular proliferation without atypia and no capsular invasion and was diagnosed with adenoma. The histologic finding in the case begs the questions of whether the histologic appearance can be considered accurate in all cases and whether histologic appearance is more appropriate for prognosis of malignant feminizing adrenal tumors than benign feminizing adrenal tumors. Andía Melero et al suggest that even in the setting of a benign histopathologic diagnosis, the clinician should be highly suspicious of a tumor secreting estrogen.^7^ Andía Melero et al did not report a Weiss score or a Ki-67 value for their patient.

Luton et al reported a case of a benign estrogen-only–secreting adrenal tumor in a female who was in her reproductive years. The patient presented with metrorrhagia as her chief complaint, and imaging revealed a mass on her adrenal gland. After resection, the patient's ovulatory cycle returned to normal, and she had no further issues at 2-month, 15-month, and 11-year follow-up. Luton et al described the histologic findings as benign, but they did not include information on special stains or Weiss score.^6^ Despite the difference in sex, we can draw parallels in the way the tumors behaved in our patient and in the patient described by Luton et al. After excision of the tumors, both patients’ symptoms improved, and neither patient had further issues at extended follow-up. This outcome suggests that for a truly benign estrogen-only–secreting tumor, excision is the only treatment necessary.

The approach to a benign feminizing adrenal tumor is similar to the approach used for other adrenocortical tumors. Evaluation of these masses should include a workup for hypercortisolism and pheochromocytoma, along with testing for sex hormones and the adrenal intermediaries listed in Table 2. Surgical resection is considered the best treatment for feminizing adrenal tumors, with laparoscopic resection being the procedure of choice if the tumor meets the guidelines for laparoscopic resection (<10 cm and not invading local tissue or lymph nodes).^5^ Surgical resection generally results in reduced gynecomastia and the other feminizing features associated with feminizing adrenal tumors. Medical therapy with tamoxifen or aromatase inhibitors and mitotane has been reported to be successful in treatment of adrenocortical carcinoma; however, no studies have compared the efficacy between these treatments.^10^

Given the sentinel event after benign histopathology in the case reported by Andía Melero et al, primary care physician follow-up of these patients is imperative. Follow-up at 6 months postoperatively and yearly thereafter should include an adequate history for identifying reoccurring symptoms, a physical examination, and trending of hormonal levels.

## CONCLUSION

Benign feminizing adrenal tumor in a male adult is a rare presentation, with prognosis and management largely unreported. More important, the ability to predict the aggressiveness or malignant potential of these tumors is uncertain. This report substantiates the importance of radiologic and histopathologic findings in the diagnosis of these tumors, as well as the need for regular postoperative follow-up with trending of hormonal levels.

## Notes

This article meets the Accreditation Council for Graduate Medical Education and the American Board of Medical Specialties Maintenance of Certification competencies for Patient Care and Medical Knowledge.
